# The Relationship of Orofacial Pain and Dental Health Status and Oral Health Behaviours in Facial Burn Patients

**DOI:** 10.1155/2021/5512755

**Published:** 2021-05-08

**Authors:** Farooq Ahmad Chaudhary, Basaruddin Ahmad, Muhammad Qasim Javed, Shaikh Shoeb Yakub, Bilal Arjumand, Asma Munir Khan, Saeed Mustafa

**Affiliations:** ^1^School of Dentistry, Shaheed Zulfiqar Ali Bhutto Medical University, Islamabad, Pakistan; ^2^School of Dental Sciences, Universiti Sains Malaysia, Kubang Kerian, Malaysia; ^3^Department of Conservative Dental Sciences, College of Dentistry, Qassim University, Al-Qassim, Saudi Arabia

## Abstract

This study aims to examine the association of orofacial pain and oral health status and oral health behaviours in facial burn patients. The participants in this cross-sectional study were randomly recruited from the Burn Care Center, Institute of Medical Sciences, Islamabad, Pakistan. An intraoral evaluation was carried out to record the DMFT and OHI-S. A self-administered questionnaire was used to collect information on sociodemographic status, brushing frequency, and dental visits. Orofacial pain during mandibular movement was assessed using the Visual Analogue Scale (VAS). Psychological status was assessed using the Generalized Anxiety Disorder Scale and Impact of Events Scale. ANOVA and simple and multiple linear regression tests were used to analyse the data. From the 90 facial burn patients included, the majority were below 34 years of age, female, single or divorced, and unemployed. The mean DMFT was 10.7, and 71% had poor oral hygiene. 56% of the participants had moderate-to-severe anxiety, and 68% had posttraumatic stress disorder. 53% of the participants had moderate-to-severe pain during mouth opening or moving the mandible with a mean score of 41.5. Analyses showed that orofacial pain was associated with less frequent brushing, irregular dental visits, greater DMFT score, and more plaque accumulation (OHI-S). It was also associated with employment status, the severity of a burn, anxiety, and stress. The treatment and management of dental and oral conditions in burn patients need judicious balance in controlling and accurate assessment of the pain and improving psychological problems in burn patients.

## 1. Introduction

Burn injuries are an example of the most debilitating physical and traumatic injuries that can potentially lead to severe physical (scars, deformities, disfigurements, and disabilities) and psychological morbidity (distress, suicidal ideation, anxiety, and posttraumatic stress) and mortality [1]. The prevalence of burn cases is declining in the developed countries, but it remains a public health concern in developing countries [2]. According to the World Health Organization (WHO), approximately 11 million cases of burn injuries, including 180,000 fatalities, occur annually worldwide and 90% of them are in low- and middle-income countries [1].

Burn patients go through a long journey towards recovery which lasts for years or decades while enduring acute, healing, and rehabilitation phases [3]. The physical complications and impairments caused by burn injuries depend upon their cause, severity, depth, and location of the burn on the body, treatment, and postburn care [3]. The major focus of burn management includes pain, infection, hypertrophic scarring, wound healing, and psychological trauma [4]. Apart from the acute posttraumatic pain, the most frequent complaint by patients is the pain caused by mechanical hyperalgesia at the affected burn area. Over time, it can lead to paresthesia, dysesthesia, loss of sensibility, and chronic pain and further worsens the psychological impact [5]. Chronic pain is another significant problem and source of discomfort and concern among burn survivors in their journey towards recovery, affecting 52% over an average of 12 years after the incident [6].

A burn to the orofacial region involving the lips and mouth has compounding impacts. The skin contraction or tightening during the healing process and scarring can lead to lip distortion and microstomia [7]. This, in turn, limits mouth opening and subsequently causes pain and discomfort while performing daily activities such as speaking, mastication, swallowing, and oral hygiene care. In a study, facial burn patients were observed to be uncomfortable and in pain when asked to open their mouth wide during the oral examination but no pain assessment was performed [8]. Pain trajectories also worsen in orofacial burn survivors who have a high level of stress, anxiety, and depression and exacerbate the previously undermined psychological conditions caused by the event [9]. Despite this understanding, the literature lacks a discussion on the impact of orofacial pain related to facial burn complications on oral condition. Hence, this study aimed to investigate the relationship between orofacial pain and oral health status and oral health behaviours in facial burn patients.

## 2. Methods

In this cross-sectional study, burn patients who visited the Burn Care Center, Pakistan Institute of Medical Sciences, Islamabad, for follow-up were randomly recruited after the ethical approval was approved from the Ethics Review Board, Shaheed Zulfiqar Ali Bhutto Medical University, Islamabad, Pakistan (F.1-23/2020/ERB/SZABMU). Participants over the age of 15 years with a burn injury to the face and neck region for more than one year and able to feed exclusively by mouth were included in this study. The participants were briefed about the purpose of the study. Written informed consent was obtained before data collection from all the participants and their parents in case of minors. All the participants underwent an intraoral examination by one investigator to record the dental (DMFT) and oral hygiene (OHI-S) status using a standard survey method by the WHO [10, 11]. The DMFT is a dental caries severity index that expresses the total number of Decayed (D), Missing (M), and Filled (F) teeth, and the total score is calculated by adding all the individual teeth scores that range from 0 to 32 [10]. The Simplified Oral Hygiene Index (OHI-S) is used to record the level of oral hygiene by assessing the debris, stains, and calculus on specific surfaces of six index teeth, with total scores ranging from 0 to 6, the latter representing the worst oral hygiene [11].

The participants also completed self-administered questionnaires that included information related to sociodemographic (age group, gender, and marital status), tooth brushing frequency (none, once, twice, or more), and dental check-ups in the past year (Yes or No). The information regarding the severity of burn injury (first-, second-, and third-degree burn) and time elapsed after that burn injury was taken from the patients' medical records.

Pain assessment was carried out by asking the participants to open the mouth widely and perform mandibular movements and then rate the pain experience by making “*X*” on the Visual Analogue Scale (VAS) [12, 13]. The VAS, a simple and commonly used analogue scale, comprises a horizontal line measuring 100 mm with “no pain” marked at 0 mm and “worst pain imaginable” at 100 mm. The pain score is the distance measured from 0 mm to the point “*X*” and ranges between 0 and 100, with a higher score suggesting greater pain intensity. The score was categorized as no pain (0 to 4 mm), mild pain (5–44 mm), moderate pain (45–74 mm), and severe pain (75–100 mm) [13]. The psychological status was assessed using the Urdu version of the Generalized Anxiety Disorder Scale (GAD-7) and Impact of Events Scale (IES) [14, 15]. The 7-item GAD-7 questionnaire assesses anxiety symptoms on a four-point Likert scale, from 0 (not at all) to 3 (almost every day). The GAD-7 score is obtained by adding all the responses and ranges from 0 to 21, whereby a greater value indicates more severe anxiety. It was categorized into four severity levels: minimal (0–4), mild (5–9), moderate (10–14), and severe (14–20) for descriptive purposes [14]. The Urdu version of GAD-7 has been validated and shown to have excellent internal constancy (Cronbach's alpha: 0.92) [14]. The 15-item IES questionnaire assesses posttraumatic stress disorder (PTSD) caused by traumatic events. The revised Urdu version of IES-R used in this study contains 7 additional items that cover three clusters of PTSD symptoms: intrusion, avoidance, and hyperarousal. The response of each item is scored on a five-point Likert scale, from 0 (not at all) to 4 (extremely). The final score ranges from 0 to 88. A threshold score >20 indicates that the individual has PTSD [15].

### 2.1. Statistical Analysis

Descriptive analysis was carried out to describe the sample and summary statistics of measures included in the study. Associations between pain related to mouth opening, jaw movement, and the factors were examined using simple and multiple linear regression. All ordinal variables were treated as continuous variables to examine the effect linear trend of exposure variables. Analyses were performed at a 5% significance level and carried out using IBM SPSS software v26.0.

## 3. Results

A total of 95 patients with a facial burn were invited to participate and 90 patients had consented and completed the oral examination and self-administrated questionnaires with a response rate of 94.7%. The sample included a high percentage of females (67%), below 25 years of age (41.1%), single, divorced, or widowed (71%), and unemployed (70%) individuals (Table 1). Most subjects had second-degree burn injuries (60%) and had the injury for 2–4 years (71%). The participants had high DMFT (mean = 10.7), and 71.1% had poor oral hygiene. More than half of the sample practiced teeth brushing once a day (61%) and did not have a dental check-up in the past year (88%). Slightly more than half of the participants had moderate-to-severe anxiety (53%) and about two-thirds had posttraumatic stress disorder (68%). About half of the participants had moderate-to-severe pain during mouth opening or moving the mandible (53%) with a mean score of 41.5.

The simple linear regression analysis showed that pain during movement of the mouth was associated with employment status, the severity of the injury, and time since the incidence (Table 2). More severe pain is associated with less frequent brushing, visits to the dentist in the past year, and greater DMFT score, plaque accumulation, anxiety, and stress.

The standardised coefficient for the DMFT in the multiple linear regression analysis was the largest compared to other factors, indicating that caries experience was the most important factor associated with pain.

## 4. Discussion

This is the first study that examines the relationship between orofacial pain related to mandibular movement and dental health status and oral health behaviours in facial burn patients and it found a significant association between them. Greater pain levels are associated with poorer dental and oral hygiene status, less frequent tooth brushing, and no visit to the dentist in the past year. The dental health status and oral hygiene were similar to a previous study on facial burn patients (mean DMFT = 10.9; poor OHI-S: 66.1%) [8]. The strong contribution of caries experience in the multivariate analysis to explain the variation in pain suggests a plausible link between orofacial pain and poor oral health conditions in patients with a facial burn injury. A burn to the facial region causes disfigurement of appearance and, in severe cases, physical impairment as a result of skin, muscles, and mucosal scarring. Tissue contraction and scarring limit mouth opening and movement and cause pain on forced opening [16]. These limit access to the oral cavity and make it extremely difficult for the patients to maintain good oral hygiene practice. Limited jaw movement also affects their chewing ability, adversely affects saliva secretion, and impairs the natural mechanism of mechanical plaque removal with the help of food during chewing [17]. A high level of oral function impairment has been linked to pain in patients with temporomandibular disorders and it could be worse in facial burn patients whose temporomandibular joint is affected [18]. Impaired chewing capability is reported to be related to TMJ pain and limitation in mouth opening and poor oral and general health status [19, 20]. Thus, the orofacial pain, coupled with poor oral health behaviours of the participants, increases the risk of developing caries. A regular visit to the dentist is highly recommended for the patients because, besides providing restorative care, dentists also can help in improving oral hygiene care such as the appropriate tooth brushing techniques for their condition and provide counselling on the caries risk factors.

This study also found that pain is more severe in older participants and those with a more recent injury, anxiety, and stress. Being older does not lower pain tolerance, likely due to ineffective pain inhibitory processes [21]. Nevertheless, the pain tends to decrease as time passes as they get used to it over time [18, 21]. The associations between pain and anxiety and stress found in the study are in line with earlier reports; the pain may lead to prolonged stress response, delay in healing, and longer recovery time [22, 23]. Depression and negative thoughts are reported in burn patients with high-intensity pain [24]. Data from this study also suggests that poor dental health status, less frequent tooth brushing, and irregular dental visits are associated with anxiety and stress (Table 3), consistent with earlier reports [25, 26].

Unemployment is another social characteristic related to higher pain expression. Chronic pain can impair working lives through a sudden change in the working environment and job loss [27]. The change in the physical appearance of these patients makes it difficult for them to find a new job or return to an old one. About 10–30% of burn survivors either do not return to work or lose the ability to work due to physical impairments injury [28]. Unemployment, brought about by disfigurement in appearance and lack of job opportunities for burn survivors who are already suffering from financial burden due to expensive and long burn care treatments, further worsens the psychological conditions of burn victims who are already distressed by the incident and its sequela [28–30]. Factors including stress from the burn incident and unemployment, education level, and having low income and older age have been associated with greater risk of pain in the jaw and face region; they are also linked to a greater risk of having poor oral conditions [8].

The findings of this study should be interpreted with caution. Limited causal inference can be made due to the cross-sectional design and associations are based only on statistical analysis. The severity of pain in relation to the need for medication and whether it truly affects oral hygiene care were not assessed. A qualitative study to assess how pain influences oral hygiene care and the extent of its effect is recommended to justify the posit. Among the greatest challenges of this study is to gain the trust of the participants who suffer from physical and psychosocial problems that make them nervous, cautious, shy, hesitant, and scared and seek sensitive information related to the dire experience and ask them to perform manoeuvres that are uncomfortable and painful.

## 5. Conclusion

This study suggests that there is a link between orofacial pain from jaw movement and oral health behaviours and poor oral conditions in facial burn patients. Factors including burn severity, time since burn injury, and psychosocial problems are also associated with orofacial pain. The findings can raise the awareness of oral health care professionals regarding the complex and multifactorial nature of dental and oral health problems in burn patients and help in preparing safe and effective strategies and practice guidelines for the care of patients with a facial burn injury. Treatment and management of dental and oral conditions in these patients require judicious balance in controlling and accurate assessment of the pain while at the same time addressing the psychological issues. Further investigations on the negative impact of orofacial pain and psychological conditions on oral health status and behaviours in burn patients are recommended.

## Figures and Tables

**Table 1:** Sociodemographic characteristics of the participants (*N* = 90).

Characteristics	Number (%)
Age	
15–24	37 (41.1)
25–34	22 (24.4)
35–44	17 (18.9)
45+	14 (15.6)

Gender	
Male	30 (33.3)
Female	60 (66.7)

Marital status	
Single/divorced/widowed	64 (71.1)
Married	26 (28.9)

Employment status	
Full-time job	18 (20.0)
Part-time job	9 (10.0)
Unemployed	63 (70.0)

Degree of burn injury	
First-degree burn	11 (12.2)
Second-degree burn	54 (60.0)
Third-degree burn	25 (27.8)

Time since burn injury	
1–2 years	16 (17.8)
2–3 years	38 (42.2)
3–4 years	26 (28.9)
4+ years	10 (11.1)

DMFT mean (SD)	10.7 (2.17)

OHI-S	
Good	8 (8.9)
Fair	18 (20.0)
Poor	65 (71.1)

Daily frequency of toothbrushing	
None	25 (27.8)
Once a day	54 (60.6)
Twice a day	9 (10.0)
More than twice	2 (2.2)

Dental check-ups	
Yes	11 (12.2)
No	79 (87.8)

GAD-7, mean (SD)	10.26 (4.18)
Normal	11 (12.2)
Mild	29 (32.2)
Moderate	32 (35.6)
Severe	18 (20.0)

IES, mean (SD)	36.3 (18.8)
No PTSD	29 (32.2)
Yes PTSD	61 (67.8)

Pain, mean (SD)	41.5 (20.6)
No pain	5 (5.6)
Mild	37 (41.1)
Moderate	39 (43.3)
Severe	9 (10.0)

**Table 2:** Association of pain with sociodemographic, burn characteristic, and psychological measures.

	Pain score Mean (SD)	SLR^1^ RC^3^ (se) *P*	MLR^2^ RC^3^ (95% CI) SC^4^ *P*
Age			
15–24	42.6 (16.8)	2.76 (1.96)	1.61 (−0.17, 3.40)
25–34	31.5 (20.7)	0.163	0.09
35–44	42.5 (21.3)		0.08
45+	53.2 (23.6)		

Gender			
Male	41.4 (21.2)	0.13 (4.64)	—
Female	43.6 (20.5)	0.97	

Marital status			
Single/divorced/widowed	40.2 (19.4)	4.68 (4.81)	—
Married	44.9 (23.6)	0.3	

Employment status			
Full-time job	15.4 (7.57)	17.3 (1.9)	4.83 (1.73, 7.93)
Part-time job	33.2 (20.1)	0.001	0.19
Unemployed	50.2 (16.1)		0.003

Degree of burn injury			
First-degree burn	10.0 (7.08)	20.2 (2.85)	5.49 (1.89, 9.09)
Second-degree burn	41.7 (14.6)	0.001	0.16
Third-degree burn	55.0 (21.3)		0.003

Time since burn injury			
1-2 years	60.5 (19.3)	−13.4 (1.98)	−2.43 (−4.90, 0.04)
2-3 years	45.9 (13.6)	0.001	−0.11
3-4 years	30.8 (18.4)		0.05
4+ years	22.1 (20.2)		Om9

Tooth brushing			
None	62.2 (12.2)	−22.5 (2.21)	−5.96 (−9.90, −2.02)
Once	38.0 (15.1)	0.001	−0.19
Twice	12.2 (11.3)		0.003
More than twice	9.0 (4.24)		

Dental visits			
Yes	22.0 (22.1)	22.1 (6.25)	—
No	44.2 (19.0)	0.001	

DMFT	41.5 (20.6)	7.40 (0.63)	3.26 (2.11, 4.42)
		0.001	0.34
			<0.001

OHI-S			
Good	13.0 (9.29)	18.5 (2.77)	—
Fair	27.8 (17.4)	0.001	
Poor	48.8 (17.4)		

GAD-7			
Normal	24.2 (19.2)	14.1 (1.78)	4.24 (1.83, 6.65)
Mild	31.1 (19.6)	0.001	0.19
Moderate	43.1 (11.4)		0.001
Severe	65.9 (11.3)		

IES-R			
No PTSD	20.7 (13.9)	30.6 (3.36)	—
Existing PTSD	51.4 (15.3)	0.001	

^1^Simple linear regression. ^2^Multiple linear regression. ^3^Regression coefficient. ^4^Standardised coefficient.

**Table 3:** Association of psychological measures with oral health behaviours and dental status.

	Tooth brushing				Dental visits		OHI-S			DMFT
	None Mean (SD)	Once Mean (SD)	Twice Mean (SD)	More than twice Mean (SD)	Yes Mean (SD)	No Mean (SD)	Good Mean (SD)	Fair Mean (SD)	Poor Mean (SD)	RC 95% CI
Anxiety	13.8 (3.87)	9.37 (3.33)	6.56 (3.32)	6.50 (3.53)	7.18 (3.02)	10.6 (4.15)	6.13 (2.16)	7.94 (3.29)	11.4 (4.02)	0.27 (0.18, 0.36)
*P* value	<0.001				<0.001		<0.001			<0.001

PTSD	53.9 (14.1)	32.3 (15.7)	16.0 (7.74)	15.0 (1.41)	28.1 (17.3)	37.4 (18.8)	17.0 (8.14)	26.0 (16.6)	41.6 (17.7)	0.08 (0.06, 0.10)
*P* value	<0.001				<0.001		<0.001			<0.001

## Data Availability

The datasets used and/or analysed during the current study are available from the corresponding author upon reasonable request.
